# Climate Change Impacts on the Marine Cycling of Biogenic Sulfur: A Review

**DOI:** 10.3390/microorganisms10081581

**Published:** 2022-08-05

**Authors:** Rebecca Jackson, Albert Gabric

**Affiliations:** 1 Coasts and Ocean Research, Oceans and Atmosphere, CSIRO, Canberra, ACT 2601, Australia; 2 School of Environment and Science, Griffith University, Nathan, QLD 4111, Australia

**Keywords:** dimethylsulfide, marine cycle, climate change, microbial community

## Abstract

A key component of the marine sulfur cycle is the climate-active gas dimethylsulfide (DMS), which is synthesized by a range of organisms from phytoplankton to corals, and accounts for up to 80% of global biogenic sulfur emissions. The DMS cycle starts with the intracellular synthesis of the non-gaseous precursor dimethylsulfoniopropionate (DMSP), which is released to the water column by various food web processes such as zooplankton grazing. This dissolved DMSP pool is rapidly turned over by microbially mediated conversion using two known pathways: demethylation (releasing methanethiol) and cleavage (producing DMS). Some of the formed DMS is ventilated to the atmosphere, where it undergoes rapid oxidation and contributes to the formation of sulfate aerosols, with the potential to affect cloud microphysics, and thus the regional climate. The marine phase cycling of DMS is complex, however, as heterotrophs also contribute to the consumption of the newly formed dissolved DMS. Interestingly, due to microbial consumption and other water column sinks such as photolysis, the amount of DMS that enters the atmosphere is currently thought to be a relatively minor fraction of the total amount cycled through the marine food web—less than 10%. These microbial processes are mediated by water column temperature, but the response of marine microbial assemblages to ocean warming is poorly characterized, although bacterial degradation appears to increase with an increase in temperature. This review will focus on the potential impact of climate change on the key microbially mediated processes in the marine cycling of DMS. It is likely that the impact will vary across different biogeographical regions from polar to tropical. For example, in the rapidly warming polar oceans, microbial communities associated with the DMS cycle will likely change dramatically during the 21st century with the decline in sea ice. At lower latitudes, where corals form an important source of DMS (P), shifts in the microbiome composition have been observed during thermal stress with the potential to alter the DMS cycle.

## 1. Introduction

Dimethylsulfide (DMS: CH_3_SCH_3_) is the most abundant form of volatile sulfur (S) in the ocean and is the main biogenic source of reduced S to the global atmosphere [1]. First Shaw (1983) [2] and then Charlson et al. [3] posited links between DMS emission to the atmosphere and the formation of atmospheric sulfate aerosols potentially affecting global climate. It was hypothesized that a temperature-driven change in marine phytoplankton growth would increase DMS emissions to the atmosphere, leading to an increase in sulfate aerosol, potentially forming more cloud condensation nuclei (CCN) and brighter clouds. This change in cloud microphysics could cool the earth’s surface and thus stabilize the climate against perturbations due to greenhouse gas warming. The proposed DMS–climate link, later called the “CLAW hypothesis” (the acronym is based on the names of the authors of the Charlson et al. [3] paper), stimulated a flurry of research in subsequent decades [4], with over 17,000 hits on the phrase “dimethylsulfide and climate” in a 2022 search on Google Scholar. The multi-layered complexity of CLAW generated research in a variety of previously unconnected and disparate disciplines (e.g., atmospheric chemistry and marine biology) and can be considered an early motivation for the development of the field of Earth System Science. Notwithstanding an enormous research effort over the last three decades, there is still no general consensus on the global validity of the original CLAW hypothesis [5,6].

The sea-to-air flux of sulfur due to DMS is estimated to be in the range of (18–31) Tg S year^−1^ [7], which constitutes about 50% of the total biogenic atmospheric sulfate burden [8,9]. In comparison, anthropogenic sulfur emissions dominate the global sulfur flux [10], and the formation of anthropogenic aerosol has caused a cooling of the global climate during the industrial period, partly offsetting the greenhouse gas warming [11]. However, this dominance of anthropogenic over natural aerosol may change during the 21st century (C) as more rigorous air pollution controls are implemented [12], with negative trends in Europe and North America already evident [13].

Once ventilated to the atmosphere, DMS is rapidly oxidized to form non-sea-salt sulfate (nss-SO_4_^2−^) and methanesulfonate (MSA) aerosols, but the efficiency of this process in producing CCN has been questioned [14]. The atmospheric chemistry of DMS is complex and still not fully understood, with novel oxidation pathways and new byproducts still being discovered [15,16].

Notwithstanding the doubts about the CLAW hypothesis, ongoing research is uncovering new ecological roles for DMS. Various marine organisms can produce intracellular dimethylsulfoniopropionate (DMSP), the precursor compound to DMS. It has been long recognized that different phytoplankton genera synthesize DMSP to varying degrees [17], with coccolithophorids and small flagellates having higher intracellular concentrations of DMSP, which was originally thought to act as an osmolyte in the algal cell. Further research has identified several other roles for DMSP, such as a response to oxidative stress in algae and corals [18], and giant clams [19], and as an osmoregulant and reduced carbon/sulfur source in marine heterotrophic bacteria [20,21,22]. In addition, DMS can act as a scavenger of reactive oxygen species (ROS) via conversion to dimethylsulfoxide (DMSO) [18,23]. Although phytoplankton have long been recognized as synthesizers of DMSP, other marine organisms such as benthic and pelagic macro-algae, corals, and sponges have also been identified as important sources of DMSP [24,25,26,27].

Recent observational studies have confirmed the importance of marine DMS emissions to atmospheric CCN [28,29] and non-sea-salt aerosol burden [30]. Additionally, strong broadscale correlations between DMS concentration and solar radiation have also been reported [31] and between solar radiation and DMS synthesis [32]. Overall, these studies lend support to parts of the CLAW hypothesis, however modeling attempts to assess the direction and magnitude of the DMS–climate feedback [33,34,35] have often led to contradictory results [36,37,38,39,40]. Other regional modeling studies indicate significant meridional variability in future DMS emissions under warming, with the strongest response simulated at high latitudes in both hemispheres [41,42,43,44].

Marine bacteria were first recognized for their role in the decomposition of organic material and the remineralization of inorganic nutrients, a role that only became fully understood during the 1980s. Azam and colleagues described a “microbial loop” in which dissolved organic matter released by phytoplankton and grazers is consumed by bacteria. Bacteria are consumed by protozoa, which are then consumed by microzooplankton that are part of the traditional grazing chain [45]. It was soon also understood that microbial organisms played key roles in the biogeochemistry of the ocean [46]. This is evidenced by the fact that the entire pelagic microbial food web, including protozoan microzooplankton, is typically 5–10 times the mass of all multicellular aquatic organisms, namely, zooplankton and fish [47].

However, the role of microbial heterotrophs in the DMS (P) cycle was not fully appreciated at the time of the original CLAW hypothesis. Indeed, it was not until two seminal papers by Simo [48] and Kiene et al. [49] that the complex role of microbes in the cycling of DMS (P) began to be better understood. The marine sulfur cycle is now considered a quintessential example of algal–bacterial interaction [50], and is the focus of intense research interest, with new components and pathways still being identified [51].

Algal intracellular DMSP can be released to the water column via a number of processes, namely, zooplankton grazing [52], exudation [53], viral lysis [54], or cell senescence. It is now well-understood that bacterioplankton are key agents in the consumption of dissolved DMSP [20], the conversion of DMSP to DMS [55], and also the consumption of DMS [56,57]. Less well-understood is the impact of elevated CO_2_ and warming on the marine microbial loop and the cycling of DMS (P). As noted by Cavicchioli et al. [58], the reciprocal effects of microorganisms on climate change and climate change on microorganisms is complex and our understanding is still incomplete. This review will examine the potential for climate change to impact on the marine bacterioplankton community structure and function, and what this may mean for future DMS (P) synthesis and emissions.

## 2. The Marine DMS Cycle

It is now recognized that organosulfur compounds both in the water and the cell play important roles in the marine food web by mediating interactions between phytoplankton and heterotrophic bacteria [59,60]. Sulfur is an essential element for marine primary production, with its algal cellular stoichiometry similar to phosphorus [61]. Thus, marine phytoplankton play a key role in the global S cycle because they can assimilate sulfate (SO_4_^2−^), which is abundant in the ocean. Sea-to-air transfer of S occurs via the ability of phytoplankton to synthesize cellular DMSP, the microbial-mediated transformation to DMS, and its subsequent emission to the atmosphere, where it has a possible involvement in climate regulation. As noted above, DMS provides the main biogenic source of reduced S to the global atmosphere. The annual global production of DMSP by deep ocean phytoplankton (excluding coastal) is conservatively estimated as 3.8 Pg C year^−1^ [62]—this is a substantial amount for a single compound, equal to approximately 10% of the total carbon fixed annually by marine phytoplankton [63]. DMSP is also critical to the structure of the marine food web as it provides a substantial source of reduced carbon and sulfur for heterotrophic bacteria [49,64] and can also be concentrated internally to serve as an osmolyte, reaching concentrations of 70 mM in some marine bacteria [21,65]. Field studies indicate that particulate DMSP concentration can span a very large range depending on the phytoplankton speciation and bloom dynamics, from 5 to >4000 nM [66,67], whereas dissolved DMSP is often present in lower concentrations (1–25 nM) and has a turnover rate of 1–129 nM d^−1^ [49].

### 2.1. Key Food Web Processes

**Figure 1:**
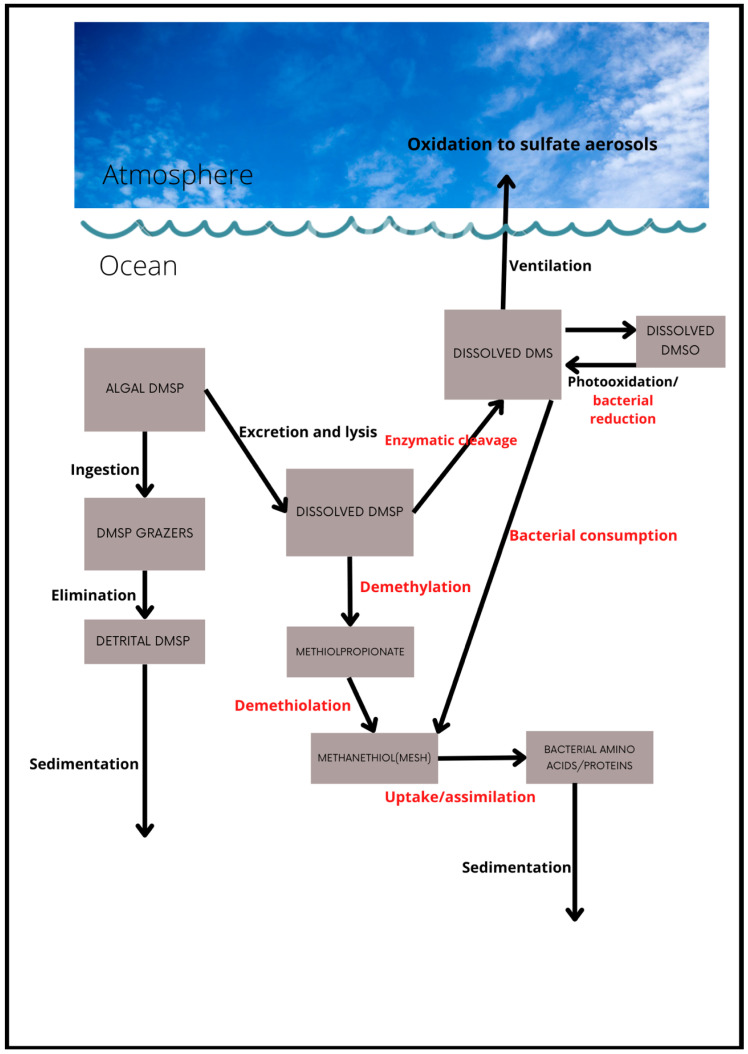
Simplified schematic representation of the dimethylsufide (DMS) and dimethylsulfoniopropionate (DMSP) cycles. Red-colored processes indicate those that are mediated by heterotrophic bacteria. DMSO: dimethylsulfoxide.

A conceptual model of the key processes in the marine cycle of DMS and its precursor compound DMSP is presented in Figure 1. Both these compounds can occur in particulate and dissolved phases. The processes included are those described for an algal DMSP source by Stefels et al. [68] and Kiene et al. [49], and emphasize the central role of heterotrophic bacteria. The main bacteria-mediated processes are colored red (Figure 1). They include the conversion of dissolved DMSP to methanethiol (MeSH; CH_3_SH), via the demethiolation pathway, or to DMS via the enzymatic cleavage pathway, and the consumption of both dissolved DMSP and DMS by bacteria. Interestingly, MeSH is also a volatile compound but its sea-to-air flux estimates are scant, although it has recently been estimated to be comparable to, albeit less than, that of DMS [69].

However, this conceptual model is only partly complete as Li et al. [70] describe a non-bioavailable pool (refractory) of dissolved DMSP, which they accounted for by retention in bacteria, but also partly unknown. Intriguingly, Liu et al. [71] report the synthesis of DMSP by bacteria in deep aphotic waters or surficial sediments, suggesting that this process is largely independent of phytoplankton production, although detrital DMSP from zooplankton grazing could also be a source in sediments [72]. Other non-algal sources of DMSP have also recently been identified, adding to the overall complexity of this cycle [24,25].

Algal intracellular DMSP is released into the water column by a variety of processes, e.g., exudation [53], cell lysis, or “sloppy” grazing [73]. Very little dissolved DMSP appears to be released by healthy, growing phytoplankton, but stressed or senescent cells and those which are infected by viruses contribute significantly to the dissolved pool [53,54]. The fate of this dissolved DMSP is crucial because either assimilation into bacterial biomass or conversion via demethylation will not lead to DMS production, thus reducing the potential for any climate regulatory effect. Moreover, it is known that only a small fraction of the DMSP that is produced is converted to DMS and thence ventilated to the atmosphere, even though, as already indicated, the contribution of DMS to the overall atmospheric sulfate burden is very significant. This raises the intriguing possibility that even a small change to the balance of these bacterially mediated processes (e.g., due to ocean warming) may cause a significant change to the DMS yield and its flux to the atmosphere.

### 2.2. Role of Heterotrophic Organisms

The uptake and assimilation of dissolved DMSP can satisfy the energy, carbon, and sulfur demands of entire marine bacterial communities [49]. A case in point is the SAR11 (*Pelagibacterales*) clade, which is of ancient evolutionary origin and comprises the most abundant and ubiquitous clade of heterotrophic marine bacteria in the oceans, estimated to make up 25% of the plankton community [74]. Tripp et al. [75] observe that assimilatory sulfate reduction is deficient in many SAR11 populations, which suggests that a requirement for exogenous reduced organic sulfur from organosulfur compounds, e.g., DMSP, methionine, and methanethiol, is widespread within the SAR11 clade.

Furthermore, O’Brien et al. [76] point out that members of the marine *Roseobacter* group (which are common in coastal waters) can establish mutualistic relationships with phytoplankton that are, in part, maintained by exchanges of DMSP. A similar sulfonate-based mutualistic interdependence between phytoplankton and SAR11 organisms has also been found in the North Pacific [77].

From the conceptual model presented in Figure 1, it is evident that the DMS yield will depend on the growth rate and biomass of the heterotrophic bacterial community [49] and on the relative importance of the enzymatic cleavage versus demethylation pathways. The demethylation pathway first converts DMSP to methylmercaptopropionate (MMPA) and subsequently to methanethiol (MeSH) or mercaptopropionate (MPA). The first step of this alternative pathway is crucial to marine sulfur emissions, because it removes a methyl group from DMSP and eliminates DMS as a possible degradation product [78]. The environmental factors that govern the utilization of one pathway over the other, and ultimately the release of DMS to the atmosphere, are uncertain. However, recent work suggests that the concentrations of DMSP that are most relevant for the bacterial production of DMS may not be the levels present in bulk seawater, but instead those existing in microscale hotspots such as near the surface of algal cells [64,79].

Measurements of DMS yield [80,81,82,83] vary widely (1–45%) depending on the region and the seasonal stage of the phytoplankton growth cycle. We note that DMS can also be produced by bacterial reduction of DMSO, the conversion by-product of DMS due to photolytic or bacterial oxidation [84], however the amount of DMS produced via this process does not seem to be significant [85].

Members of the *Roseobacter* group, a ubiquitous group of marine bacteria, are well-known MeSH producers [86], and in waters rich in these bacteria, consumption of DMSP via demethylation can be an order of magnitude larger than DMS production via cleavage. Kiene et al. [49] posited that bacterioplankton will prefer the demethylation/demethiolation over the lyase pathway at low dissolved DMSP concentrations. This is because this pathway provides more energetic benefits, and it is a relatively economic way to assimilate reduced sulfur. At higher dissolved DMSP concentrations, the DMSP that is not assimilated is then available to the cleavage pathway. It is thus likely that DMS yield through the enzymatic cleavage pathway will increase if bacterial sulfur demand is met with excess sulfur [82], which can occur during the later stages of a phytoplankton bloom [9].

### 2.3. Impacts of Changes to Ocean Temperature and pCO_2_

The global oceans face multiple CO_2_-driven impacts in the coming decades, including ocean acidification (OA), warming, deoxygenation, and loss of sea ice cover [87], all of which will likely affect the carbon budget of the biosphere which depends largely on the balance between the uptake of carbon by phytoplankton photosynthesis and its remineralization by heterotrophs [88]. Warming of the ocean will directly and indirectly impact the growth rates of phytoplankton, potentially altering global primary production and leading to major shifts in the functional trait composition of marine phytoplankton communities [89,90]. The directionality of the productivity response, either positive or negative, will depend on the temperature sensitivity of phytoplankton both within and across species [90], with higher temperatures often leading to a shift towards smaller species such as cyanobacteria [91,92,93]. Similarly, changes in temperature have been found to regulate the metabolic rates of bacteria in marine environments [94]. Thus, the balance between autotrophic production and heterotrophic respiration is profoundly affected by the environmental temperature [95]. However, attempting to describe the response of bacteria to temperature is challenging since other environmental factors that covary with temperature, such as nutrient concentration or primary productivity, may themselves have a larger effect on bacterial properties [96]. Despite the complex interactions between temperature, substrate availability, cell size, and abundance in the ocean, strong apparent increases with temperature in the abundance of picoplankton (<2 μm in diameter), which includes all free-living heterotrophic bacteria, have consistently been reported across different aquatic ecosystems [97]. However, the combined impacts of temperature and pCO_2_ increases appear to confound the situation, with decreases in phytoplankton biomass combined with increases in bacterial production reported in the Arctic [98].

Over the last two decades, numerous studies have explored the effect of climate change on the broad structure and functioning of the marine food web and the possible changes in net primary production (NPP). This has been attempted through both modelling and mesocosm experiments [99,100,101,102,103,104]. However, owing to computational constraints, temperature sensitivities of recycling processes and especially the microbial loop are ignored in most global models [104,105], notwithstanding that it has long been recognized that bacteria dominate the abundance, diversity, and metabolic activity of the ocean [106] and that heterotrophic processes such as bacterial degradation are known to be temperature-dependent [107]. Brewer and Peltzer [108] provide an excellent review of the broader aspects of this issue in the context of ocean de-oxygenation and bemoan the general neglect of the temperature dependence of microbial decomposition rates in the literature.

### 2.4. Modeling

Prognostic DMS models have been under development since the early 1990s [109]. Most of the initial efforts were built on food web compartment model approaches [110], and focused on understanding the relationship between phytoplankton bloom dynamics and DMS (P) production in specific regions, e.g., the Arctic [111,112], the Southern Ocean [113], the North Atlantic [114], and the Northwest Pacific [115]. Most of these modeling attempts have been to some degree constrained by limited data on the model parameters. Although sensitivity analyses and other techniques can assist with model calibration at the local scale [116], a major problem in applying such models at a global scale is the site-specific nature of parameter calibration in the field.

The difficulty of applying DMS prognostic models at the global scale has prompted efforts to develop globally applicable diagnostic schemes that predict DMS seawater concentration based on variables such as surface chlorophyll and light, which can be retrieved using satellite remote sensing algorithms [62,117,118,119,120]. These schemes are based on linear regression techniques to estimate DMS concentrations using one or more predictors. Some schemes have demonstrated reasonably good performance at the global scale, but their predictive power is generally less at the regional scale [121]. Currently, none of the global schemes have been accepted universally, with the main deficiencies related to regional biases in remotely sensed chlorophyll (which cause underestimation of DMS in the Southern Ocean) and the inability to reproduce observed variability in “DMS hotspots” such as the northeast sub-Arctic Pacific [122].

Notwithstanding the lack of a globally applicable DMS prediction algorithm [123], there have been numerous attempts to simulate the impact of climate change on future DMS seawater concentration and flux to the atmosphere [37,41,42,124,125,126,127]. Typically, these studies have coupled a global climate model with either a biogeochemical DMS model or empirical prediction algorithm, where the relevant future forcings such as sea surface temperature (SST), surface wind speed, and ocean mixed layer depth drive the future change in DMS seawater concentration.

Bock et al. [39] examine trends of surface ocean DMS concentration and flux of four Earth system models (ESMs: CNRM-ESM2-1, MIROC-ES2L, NorESM2-LM, and UKESM1-0-LL) over the recent past (1980–2009) and into the future, using Coupled Model Intercomparison Project 6 (CMIP6) simulations. The four ESMs disagree on the sign of the trend of the global DMS flux from 1980 onwards, with two models showing an increase and two models a decrease. At the global scale, these trends are dominated by changes in surface DMS concentrations in all models, irrespective of the air–sea flux parameterization used. Three of the models consistently show that changes in DMS concentrations are correlated with changes in marine productivity; however, marine productivity is poorly constrained in the current generation of ESMs. In contrast, a consensus is found among all models at polar latitudes, where an increasing trend is predominantly driven by the simulated retreat in sea-ice extent, however, the magnitude of this trend between models differs by a factor of three. Given the variety of biogeochemical model formulations used, it is not surprising that the simulated changes in DMS under warming vary significantly. In summary, there is no agreement in the literature with regards to the sign and amplitude of the trend in DMS concentration and flux in the future [128].

As indicated above, it is likely that the simulated variability in future DMS trends is related to the inability of current ESMs to accurately project future changes in marine primary production. Depending on whether the ESM formulation has biological processes that are temperature-dependent or not, simulated NPP can either increase or decrease under projected climate change [129]. Indeed, a comparison of nine marine ecosystem model projections of changes in global marine NPP showed quite large uncertainties, especially at low latitudes [130]. Most global ESMs used for carbon sink projections in the Fifth Assessment Report of the Intergovernmental Panel on Climate Change (IPCC AR5) ignored the impact of warming on organic carbon remineralization and the biological carbon pump [105]. Segschneider and Bendtsen [104] quantified the impact of including temperature-dependent remineralization (TDR), modifying the CMIP5 model MPI-ESM and its marine biogeochemistry model HAMOCC5.2, and projected an ~0.18 PgC year^−1^ reduction in ocean carbon uptake by 2100 under the high-emission scenario RCP8.5. The inclusion of more complex ecological parametrizations in the ESM simulates a more modest decline in the ocean carbon sink capacity of ~0.06 PgC year^−1^ during the 21st century [105], compared to the estimated 2020 ocean carbon sink uptake rate of 3.0 ± 0.4 PgC year^−1^ [131].

### 2.5. Laboratory Experiments

Over the past decade, several mesocosm and ship-board bioassay experiments have examined the impact of future increased temperature and pCO_2_ level on the DMS (P) cycle by perturbing either one or both variables simultaneously. Avgoustidi et al. [132] looked at the change from an approximate doubling of pCO_2_ (700 ppm) in *Emiliania huxleyi* and found that DMS production was roughly halved, which they attributed to a change in intracellular DMSP at lower pH values. Spielmeyer and Pohnert [133] analyzed DMSP in separate cultures of diatoms (*Thalassiosira pseudonana*, *Phaeodactylum tricornutum*) and a prymnesiophyte (*Emiliania huxleyi*) under the influence of increased temperature, by 6 °C, and elevated CO_2_ to 790 ppmv. The diatoms and prymnesiophytes revealed opposite trends for DMSP. In diatoms, increased CO_2_ and temperature led to decreased DMSP concentrations, however elevated levels of this metabolite under the influence of these parameters were observed for *Emiliania huxleyi.*

Arnold et al. [134] also report measurements of DMSP and DMS concentrations in pH-stated cultures of *Emiliania huxleyi*. Four different environmental conditions were tested: ambient, elevated CO_2_ (+CO_2_), elevated temperature (+T), and elevated temperature and CO_2_ (+TCO_2_). In comparison to the ambient treatment, average DMS production was about 50% lower in the +CO_2_ treatment. Importantly, temperature had a strong effect on DMS production, and the impacts outweighed the effects of a decrease in pH, with a more than doubling of intracellular DMSP.

Park et al. [135] conducted a series of perturbation experiments in Korean coastal waters and found that at both ambient temperature and ~+2 °C warmer, an increase in pCO_2_ (160–830 ppm) favored the growth of large diatoms, which outcompeted other phytoplankton species and reduced the growth rate of smaller, DMSP-rich dinoflagellates. This decreased the grazing rate of heterotrophic dinoflagellates, resulting in reduced DMS production via grazing activity, and highlights the influence of community composition changes on future DMS production under warming.

Hopkins and Archer [136] conducted 96 h bioassay ship-board pCO_2_ perturbation experiments in NW European waters and found that the changes induced consistent, marked increases in DMS and decreases in DMSP, in contrast to results from longer-term mesocosm experiments. Bacterially mediated DMS processes appeared to be insensitive to ocean acidification, with no detectable effects on dark rates of DMS consumption and gross production and no consistent response seen in bacterial abundance [136].

Li et al. [137] conducted a similar experiment with a culture of the dinoflagellate *Amphidinium carterae* to investigate the effects of elevated CO_2_ concentration and temperature on the growth and production of DMSP and DMS, however they found no significant effects on the concentrations and cell-normalized concentrations of DMSP or DMS. Saint-Macary et al. [138] observed a reduction in DMSP at warmer temperatures which was associated with changes in the phytoplankton community, and in particular with small flagellate biomass. A smaller decrease in DMS concentration was measured in the treatments relative to other studies.

Significant positive correlations were found between bacterial production and concentrations of DMS in Canadian waters during autumn. The 13-day mesocosm experiments were characterized by blooms of diatoms, with the results pointing to temperature-associated enhancement of bacterial DMSP metabolism and large increases to DMS (>200%), negating any decreases due to acidification [139]. However, later work by the same group of authors in the same Canadian waters during summer found that doubling and tripling the pCO_2_ resulted in a 15% and 40% decline in average concentrations of DMS compared to the control. Results from S-35-DMSPd uptake assays indicated that neither concentrations nor microbial scavenging efficiency of dissolved DMSP was affected by increased pCO_2_. However, their results show a reduction of the mean microbial yield of DMS by 34% and 61% in the 2 × pCO_2_ and 3 × pCO_2_ treatments, respectively [140].

Clearly, the results of these perturbation experiments are varied and appear to sensitively depend on the phytoplankton groups present, the ocean region examined (polar or temperate), and the perturbation applied. Subtle interactions are also evident, which can lead to counter-intuitive results. For instance, in studies of natural communities that are dominated by the DMSP-rich species *Emiliania huxleyi*, it would seem likely that the adverse effect of increased pH on the growth of this calcifying organism would also result in a negative impact on the overall production of both DMSP and DMS [141], but this is not always the case, as indicated by the results of Arnold et al. [134].

The range of different results in these experiments makes it difficult to present a synthetic view of the impact of temperature and/or pCO_2_ changes on future DMS (P) production. It is apparent that the results are sensitive not only to the phytoplankton functional group present but also to the length and type of experiment (mesocosm versus bioassay) and thus the potential for shifts in the community composition to occur.

## 3. Projected Ocean Warming and Acidification

### 3.1. Global CMIP6 Climate Projections

The Coupled Model Intercomparison Project Phase 6 (CMIP6) provides climate projections for a range of scenarios to understand the past, present, and future climate. For a better understanding of the impact of the projected climate change on SST, mixed layer depth (MLD), and pH, these are examined for both a contemporary (2001–2020) and end of century (2081–2100) climate, as simulated by CMIP6 models under a SSP2-4.5 and SSP5-8.5 Shared Socioeconomic Pathway (SSP). The possible impact of ocean warming and acidification on DMS (P) production and cycling is then discussed below.

Model output for monthly mean SST (°C) and MLD (m) was obtained from the Australian Community Climate and Earth-System Simulator-Coupled Model version 2 (ACCESS-CM2) [142] for the CMIP6 historical, SSP2-4.5, and SSP5-8.5 experiments. Monthly mean pH was also obtained from the Geophysical Fluid Dynamics Laboratory Earth System Model version 4 (GFLD-ESM4) [143].

The historical simulations run to 2014, with solar variability, volcanic aerosols, and anthropogenic-driven changes in atmospheric composition (GHG and aerosols) forced by datasets that are largely based on observations [144]. The contemporary climatology was extended from 2014 to 2020 by including an average of the CMIP6 model output for the SSP2-4.5 and SSP5-8.5 scenario experiments. For the future SSP scenarios, variables are simulated from 2015 onwards under the respective emissions trajectory. For SSP2-4.5, a medium positive radiative forcing (+4.5 Wm^−2^) is expected by 2100, assuming a shift towards renewable energy and sustainable development [145]. For SSP5-8.5, a high positive radiative forcing (+8.5 Wm^−2^) is expected by 2100, assuming that development will be largely driven by fossil fuels [146]. Data were obtained for the r1i1p1f1 ensemble and are available from the Earth System Grid Federation (https://esfg-node.llnl.gov/search/cmip6 (accessed on 19 June 2022)).

#### 3.1.1. Ocean Warming

**Figure 2:**
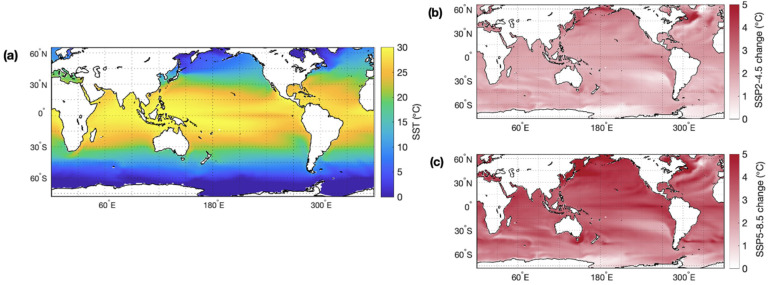
(**a**) Present-day annual mean sea surface temperature (SST) and the change in SST by the end of this century, as simulated by CMIP6 climate models for climate under two emission scenarios: (**b**) SSP2-4.5 and (**c**) SSP5-8.5.

Ocean warming poses a serious threat to many marine ecosystems. Anthropogenic GHG emissions trap solar radiation within the Earth’s atmosphere, leading to an accumulation of excess heat energy. More than 90% of this excess heat is stored in the ocean, leading to a 0.5 °C rise in global mean SST over the past 40 years [147]. By the end of this century, global mean SST is predicted to increase by a further 1.8 and 3.2 °C under a SSP2-4.5 and SSP5-8.5 climate, respectively (Figure 2).

**Figure 3:**
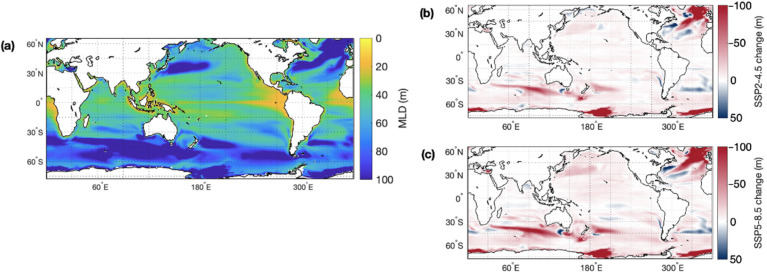
(**a**) Present-day annual mean mixed layer depth (MLD) and the change in MLD by the end of this century, as simulated for climate under two emission scenarios: (**b**) SSP2-4.5 and (**c**) SSP5-8.5.

Temperature impacts the physiology of marine organisms, ocean currents, mixing, and the availability of nutrients in the ocean. Rising SST is predicted to contribute to a stratification of the upper ocean layers and an overall reduction in global mixed layer depth, particularly in the Southern Hemisphere mid–high latitudes and North Atlantic Ocean, where present-day MLD is deepest (Figure 3). Reduced vertical mixing in a more stratified ocean will likely have impacts on the marine sulfur cycle by reducing nutrient concentrations and limiting productivity in the photic zone.

#### 3.1.2. Ocean Acidification

**Figure 4:**
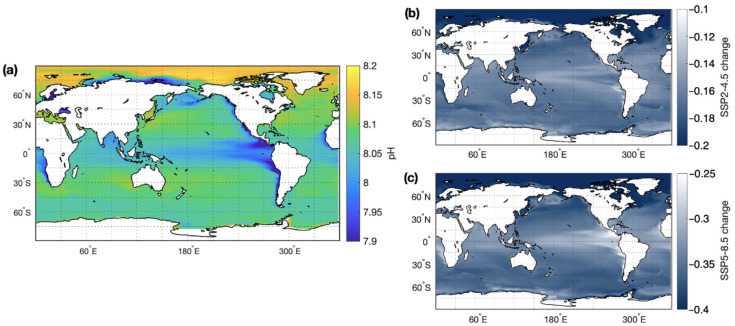
(**a**) Annual mean pH and the change in pH by the end of this century, as simulated for climate under two emission scenarios: (**b**) SSP2-4.5 and (**c**) SSP5-8.5.

Ocean acidification occurs due to increased absorption of atmospheric CO_2_. Dissolution of CO_2_ forms carbonic acid, which readily dissociates into bicarbonate ions and protons, thereby reducing pH. Current anthropogenic CO_2_ emissions are 10.1 ± 0.5 Gt C year^−1^ [148]. Approximately 30% of anthropogenic CO_2_ is absorbed by the ocean and has led to a decrease in global mean ocean pH of 0.1 units over the past 100 years [147]. Despite a reduction in CO_2_ emissions by the end of this century under an SSP2-4.5 scenario, global mean ocean pH is predicted to decline by −0.18 (See Figure 4). The decline in pH is more extreme under the SSP5-8.5 scenario (−0.36), where little or no effort is made to constrain anthropogenic emissions [146]. Ocean acidification is already impacting on the physiology of marine species, particularly those which are dependent on bioavailable carbonate such as coral reefs.

### 3.2. Impacts on DMS Cycling in the Polar Oceans

The polar oceans contribute a significant proportion of global DMS emissions and are warming more rapidly than lower latitudes. Factors such as rapid sea ice retreat in the Arctic [149] and the presence of DMSP-rich species such as *Phaeocystis antarctica* in the Southern Ocean [150] contribute to the potential for major changes in the structure of polar ecosystems and DMS production as warming unfolds [151]. The role of heterotrophic bacteria in the polar regions has been unclear as it was thought that they would be less active because of low temperatures, however Kirchman et al. [152] suggest that the lower activity is partly temperature-driven but mainly due to lower dissolved organic matter (DOM) inputs, a situation which could be exacerbated by increased stratification under warming. In a decade-long study at a highly productive site (Palmer Station) in the Western Antarctic Peninsula (WAP) site, Kim and Ducklow [153] report that bacterial production (BP) was only 4% of the primary production (PP), consistent with the low BP:PP ratios observed in other studies of polar waters. The degree of bottom-up control on bacterial abundance was moderate and relatively consistent across entire growing seasons, suggesting that bacteria in the coastal WAP are under DOC limitation. Temperature also influenced BP rates, though its effect was weaker than the supply of DOC, which is consistent with the findings of Kirchman et al. [152].

The polar oceans are characterized by high dissolved inorganic carbon (DIC) concentrations and a low carbonate system buffering capacity, mainly due to the increased solubility of CO_2_ in cold waters [154]. This makes these polar regions particularly susceptible to the impacts of OA [155], although there is also evidence of regional variability between the Arctic and Southern Ocean due to differing micro-nutrient regimes [156]. Although quite limited, analyses of the effect of OA on DMS production suggest that OA could significantly decrease the algal biomass and inhibit DMS production during the seasonal phytoplankton bloom in the Arctic [157,158].

In contrast, Hopkins et al. [159] report findings from seven summertime ship-board microcosm experiments undertaken in a variety of locations in the Arctic Ocean and Southern Ocean, which reveal no significant effects of short-term OA on the net production of DMS by planktonic communities. In a follow-up meta-analysis, Hopkins et al. [160] examined experiments in both temperate and polar waters and found clear regional differences in the DMS response to OA, leading them to conclude that future temperate oceans could be more sensitive to OA, while perhaps surprisingly, DMS emissions from the polar oceans may remain relatively unchanged.

### 3.3. Impacts on the Tropical Ocean and Coral Reefs

Ocean warming and acidification pose serious threats to tropical coral reef ecosystems, with impacts already evident through more frequent and severe coral bleaching events [161,162,163], reduced coral cover and rates of larval recruitment [164], and a decline in coral calcification and growth rates [165].

**Figure 5:**
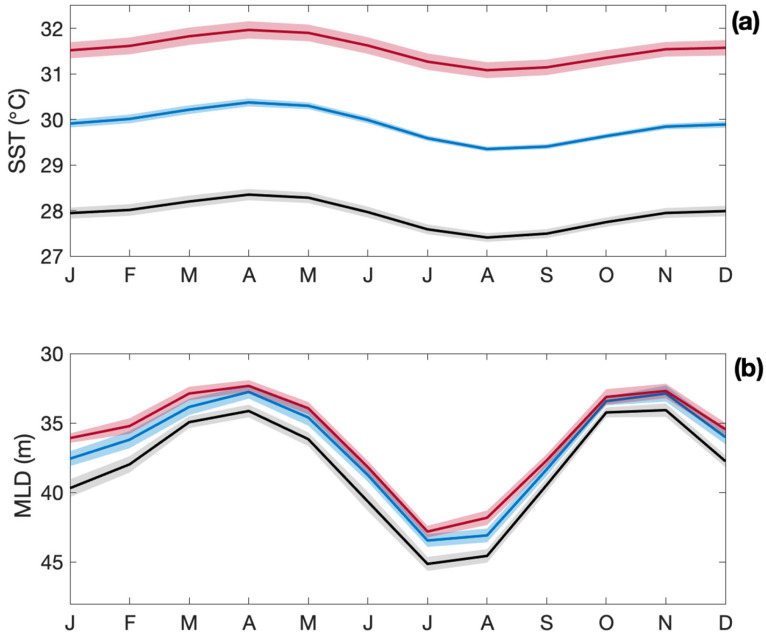
Climatology (±2 SE) of (**a**) SST and (**b**) MLD for a contemporary (black) and end-of-century climate modeled under two emission scenarios: SSP2-4.5 (blue) and SSP5-8.5 (red) scenarios for the tropical ocean (20° N–20° S).

Reef-building Scleractinian corals have relatively narrow temperature tolerance ranges [163,166,167], and many already live in regions where SST is close to their thermal tolerance thresholds, meaning that increases in SST as small as 0.5 °C could be sufficient to cause coral bleaching [168]. It is predicted that annual severe coral bleaching events will affect more than 95% of tropical coral reefs by 2050 [169]. At 1.5 °C above pre-industrial SST, coral thermal refugia zones and recovery periods are predicted to decline [170,171] and ongoing coral reef degradation may become unavoidable. By the end of this century, CMIP6 models predict tropical SST to increase by a respective 2.0 and 3.6 °C under an SSP2-4.5 and SSP5-8.5 scenario (Figure 5a), with likely detrimental impacts on the coral reef ecosystem structure. Mixed layer depths, which are already shallow in the tropical ocean, shoal further under warming scenarios (Figure 5b).

Coral reef ecosystems are important regional sources of dissolved and atmospheric sulfur, emitting an estimated 0.05–0.08 Tg year^−1^ of DMS (equivalent to 0.025–0.04 Tg year^−1^ S) [172]. Reported concentrations of atmospheric DMS (DMS_a_) in the Great Barrier Reef (GBR), Australia, average approximately 30 ppt in winter and 100 ppt in summer [173,174], and can exceed 500 ppt over coral reefs that have been exposed to the atmosphere during low tide [174,175]. On one occasion at Heron Island in the southern GBR, DMS_a_ concentration reached 1149 ppt when the coral reef flat was thermally and osmotically shocked by rainfall [174]. These concentrations are up to two orders of magnitude higher than the background ocean, meaning that coral reefs are important hotspots of marine sulfur. However, the future contribution of coral reefs to the dissolved and atmospheric sulfur pool will depend on the ability of corals to acclimate to their rapidly changing environment.

DMSP biosynthesis and cleavage to DMS is upregulated in corals in response to thermal and irradiance stress [175,176,177]. When high temperatures combine with elevated light levels, the photosystems of zooxanthellae and free-living algae can become photo-inhibited and produce ROS [18,178,179,180]. If conditions persist, ROS accumulate and cause oxidative damage in the coral holobiont [167,180,181]. To cope with oxidative stress, corals may change the composition of their microbiome (e.g., zooxanthellae switching or shuffling) to enhance temperature tolerance [182] or may expel zooxanthellae and become bleached [167,181,183]. DMS (P) plays an important role in mitigating oxidative stress in corals [178] and marine algae [18] by scavenging ROS and forming DMSO.

Shifts in the coral holobiont microbiome can result in a change in DMS (P) production [184,185]. DMS (P) concentrations vary between endosymbiont types, with more temperature-sensitive types (e.g., clade C) typically containing higher concentrations than temperature-tolerant types (e.g., clade D) when exposed to elevated temperatures [182,186]. However, tolerance thresholds vary between coral and endosymbiont species [187], and other factors such as assemblage complexity [19] can influence DMS (P) production in coral reefs.

If corals can withstand rising ocean temperatures through natural means, such as the recruitment of temperature-tolerant endosymbionts [182,184], DMS production and emissions from coral reefs may increase in the future. However, observational studies have shown that seawater surface and atmospheric DMS concentrations in the GBR increase with SST, until SST approaches coral thermal tolerance thresholds [175,188]. Beyond this threshold, ambient DMS concentrations can decline [175,185,189], possibly due to an upregulation of the coral and algal antioxidant response discussed above. Therefore, if corals cannot acclimate, DMS concentrations and emissions may decline due to an upregulation of the coral antioxidant response [178], or due to increased coral bleaching and mortality leading to reduced coral cover [164].

Exacerbating the impacts of ocean warming, ocean acidification has already led to a decline in the bioavailability of carbonate ions [190], limiting calcification and growth of calcareous organisms. In the GBR, skeletal density and linear extension rates of massive *Porites* corals have declined by 0.36% and 1.02%, respectively [165]. Although the impacts of ocean acidification on the coral reef sulfur cycle are unclear, a decline in coral cover will likely shift the balance of coral–algal dynamics in reef ecosystems.

As for ocean regions, algal and microbial communities play an important role in the production and cycling of DMS (P) in the coral holobiont and in coral reef waters [177]. Increasing SST and enhanced stratification (Figure 5) may limit nutrient availability and phytoplankton productivity in the GBR. However, GBR waters are predicted to become more eutrophic with increasing inputs of nitrogen and phosphorus from agricultural runoff and soil erosion [191,192,193,194]. Improving water quality is a key commitment of the Australian Government’s Reef 2050 Long-Term Sustainability Plan.

Further, corals and algae compete for light, nutrients, and space in the reef matrix. Predicted declines in coral cover due to ocean warming, acidification, predation, and declining water quality may lead to an increase in algal biomass in coral reefs. Increased algal biomass is often observed after mass coral bleaching and mortality events [195], impeding coral growth and reducing opportunities for new coral establishment [196]. An increase in alternative sulfur sources (e.g., through riverine inputs) reduces sulfur limitations and thus the need for microbial DMSP demethylation, thereby increasing DMS yields via the alternative cleavage pathway [22].

## 4. Conclusions

Ecological interactions between marine bacteria and phytoplankton are now recognized as playing a central role in the ocean’s major biogeochemical cycles. How these interactions may be modified as a result of climate change is the focus of this review, which examined the role of heterotrophic bacterioplankton in the cycling of a suite of organo-sulfur compounds, such as DMS. This volatile compound and its precursor DMSP are ubiquitous in the oceans, being synthesized by a large range of marine organisms from algae to corals. After ventilation, DMS constitutes the largest source of natural sulfur to the atmosphere and is thus also an important contributor to the atmospheric burden of sulfate aerosol with key relevance to the Earth’s radiative balance and climate.

Due to its synthesis by numerous marine organisms, DMSP and its related compounds are an important source of DOM for bacterial heterotrophy in the surface ocean, where it has been estimated to contribute up to 100% of the sulfur requirements of some important marine bacteria groups. There is growing evidence that sulfonates serve as an ecologically important currency for nutrient and energy exchange between autotrophs and heterotrophs, highlighting the importance of organo-sulfates in regulating ecosystem function. This suggests that climate change impacts on the synthesis and cycling of DMS (P) could have important future consequences for the structure of the marine food web.

However, perturbation experiments seeking to understand the impact of warming and OA on the synthesis of DMS have so far yielded equivocal results. The range of different results makes it difficult to provide a synthetic view of the impact of temperature and/or pCO_2_ changes on future DMS (P) production. It is apparent that the results are sensitive not only to the phytoplankton functional group present but also to the length and type of experiment conducted.

Although numerous attempts have been made to numerically model the impact of warming on the DMS (P) cycle, there is no general agreement in the literature with regards to the sign and amplitude of the future trend in DMS concentration and sea-to-air flux. Importantly, these efforts are also constrained (due to current knowledge of the process and computational limits) by a lack of an explicit temperature-dependent parameterization of the key bacterial-mediated processes, such as demethylation and enzymatic cleavage. Considering the critical roles DMS and its precursor DMSP appear to play in supplying carbon and sulfur to marine heterotrophs, future efforts should focus on informing the many uncertainties still apparent in the literature regarding the ecological role of these compounds, and especially the nexus between future CC impacts on marine bacteria and the possible resultant changes in the marine DMS (P) cycles.

## Data Availability

The CMIP6 model output used in this analysis [142,143] is available from the Earth System Grid Federation (https://esfg-node.llnl.gov/search/cmip6/ (accessed on 19 June 2022)).
